# An experimental study of messages communicating potential harms of electronic cigarettes

**DOI:** 10.1371/journal.pone.0240611

**Published:** 2020-10-21

**Authors:** Daniel Owusu, Zachary Massey, Lucy Popova

**Affiliations:** School of Public Health, Georgia State University, Atlanta, Georgia, United States of America; Medical University of South Carolina, UNITED STATES

## Abstract

There has been an upsurge of e-cigarette use in the United States in recent years. While e-cigarettes may contain lower levels of toxic chemicals than combusted cigarettes, they still pose serious health hazards, including increased risk for heart and respiratory disease. Despite these risks, public awareness of the health harms of e-cigarettes remains low. Thus, it is important to educate the public about the potential harms of e-cigarettes. This study took themes commonly found in antismoking messages and used them to develop messages about harms of e-cigarettes. A national sample of 2801 current smokers and nonsmokers (aged 18+ years) were randomized to view one of four e-cigarette messages (harmful effect of chemicals, uncertainty about ingredients, distrust of big tobacco, or cost of vaping) or a control message (bottled water ad). Participants’ reactions to the messages and behavioral intentions were assessed immediately following the exposure. MANOVA examined effects of the messages on blocks of the outcome variables and univariate analyses estimated adjusted means for each experimental condition for each outcome. The message about harmful chemicals was perceived as the most informative and effective and elicited the highest levels of negative emotions (*Ps*<0.05). However, on measures of actual effectiveness, the other messages performed equally well. Specifically, messages with different themes (harmful chemicals, uncertainty about ingredients, anti-industry, or financial cost) increased perceived risk of e-cigarettes, support for e-cigarette control, and lowered self-exempting beliefs and intentions to use e-cigarettes (*Ps*<0.05). Themes commonly used in anti-smoking messages may be effective in educating the public about the potential harm of e-cigarettes. The observed differential effects of the messages suggest the need to use multiple themes in a public education campaign about e-cigarettes.

## Introduction

In recent years, there has been an increase in the use of electronic cigarettes (e-cigarettes) in the United States. In 2010, only 1.8% of adults reported having ever used e-cigarettes [1] compared to 15.3% in 2016 [2]. During the same time, e-cigarette ever use increased among never-smokers from 0.3% in 2010 [1] to 5.7% in 2016 [2], when 15% of ever e-cigarette users were never smokers. Alarmingly, the increase in e-cigarette use has been more dramatic with youth, with current use jumping from 1.5% in 2011 to 27.5% in 2019 among 12^th^ graders [3]. From 2017 to 2018, there was a 48.5% increase in current e-cigarette use for middle school students (from 3.3% to 4.9%) [4], and a 46.2% increase in current use for young adults (from 5.2% to 7.6%) [5]. This rise in e-cigarette use threatens to detract from other successful efforts to reduce overall tobacco use in the US [4].

While e-cigarettes contain lower levels of harmful chemicals than combusted cigarettes [6–8], they still pose health hazards. For instance, they may increase blood pressure and the risk for myocardial infarction [8–11] and damage DNA and increase cell deaths [8–13]. E-cigarettes may also increase the risk for lung diseases, including asthma, chronic obstructive pulmonary disease, and acute-onset bronchiolitis obliterans [14–16]. Despite this mounting scientific evidence, public awareness of health harms from e-cigarettes remains low [17], which may increase misconceptions about the risk of using these devices [18]. For instance, a recent study reported that only about 16% of the participants were aware of e-liquid toxicity [19]. Tobacco and e-cigarette industries aggressively promote e-cigarettes as healthy or less harmful than combustible cigarettes [20], and this marketing contributes to low perceived risks of e-cigarettes [21]. Therefore, the health risks of e-cigarettes and the comparative risk of e-cigarettes (risk of e-cigarettes relative to combusted cigarettes) need to be communicated to the public to help informed-decision making.

In the nascent field of communicating about e-cigarettes, existing studies have predominantly focused on the comparative risk of e-cigarettes [21–28] with less attention paid to the absolute risk of e-cigarettes. With the implementation of a national youth e-cigarette use prevention campaign [29], and the FDA’s deeming rule requiring e-cigarette manufacturers to include warning statements about nicotine [30, 31], a few studies have assessed impacts of e-cigarette health warnings, predominantly industry-style warning labels, and reported mixed findings. While some studies found that health warning labels increased risk perception and reduced intention to try e-cigarettes [24, 32, 33], others found that the addiction warning did not increase or showed an inconsistent effect on risk perception of e-cigarettes [34, 35]. Research on e-cigarette prevention messages and campaigns is nascent [36–38], but indicates that they might be effective in increasing perceived risk and reducing use intentions [38]. Thus, research is needed to identify messages that effectively communicate potential harms of e-cigarettes in addition to FDA-mandated warning, similar to antismoking communications.

Antismoking messages, such as warning labels and media campaigns, have been found to increase beliefs about harms of tobacco, negative attitudes towards smoking, intentions not to smoke, and intentions to quit smoking [39–42]. Antismoking messages often employ fear appeals [43, 44] by presenting health facts intended to arouse negative emotional responses that will motivate healthy behaviors. Another effective strategy is to highlight the deceptive and unethical practices of the tobacco industry [45]. Because of their success, the strategies used in framing antismoking communication can be used to communicate about potential harms of e-cigarettes. However, little is known about the effectiveness of this type of communication when applied to e-cigarettes. To fill this gap, we employed themes used in antismoking communications to develop different types of e-cigarette messages and tested them in a randomized controlled experiment with an adult sample from the US. We aimed to comprehensively assess reactions, perceptions, and behavioral intentions following the exposure to these messages. We relied on the Message Impact Framework, which specifies different types of outcomes (message reactions, attitudes, beliefs, and intentions) that serve as antecedents to behavioral change [40].

## Method and materials

Working in collaboration with a social marketing agency, we developed 4 print messages that focused on communicating harms of e-cigarette use. The messages were created based on the review of existing educational campaigns about e-cigarettes from public health agencies, such as California’s *Still Blowing Smoke* campaign [46], and extant literature on constituents and health effects of e-cigarettes. We also used insights from our qualitative study that explored smokers’ interpretation of messages comparing the risks of cigarettes and e-cigarettes [18].

**Fig 1:**
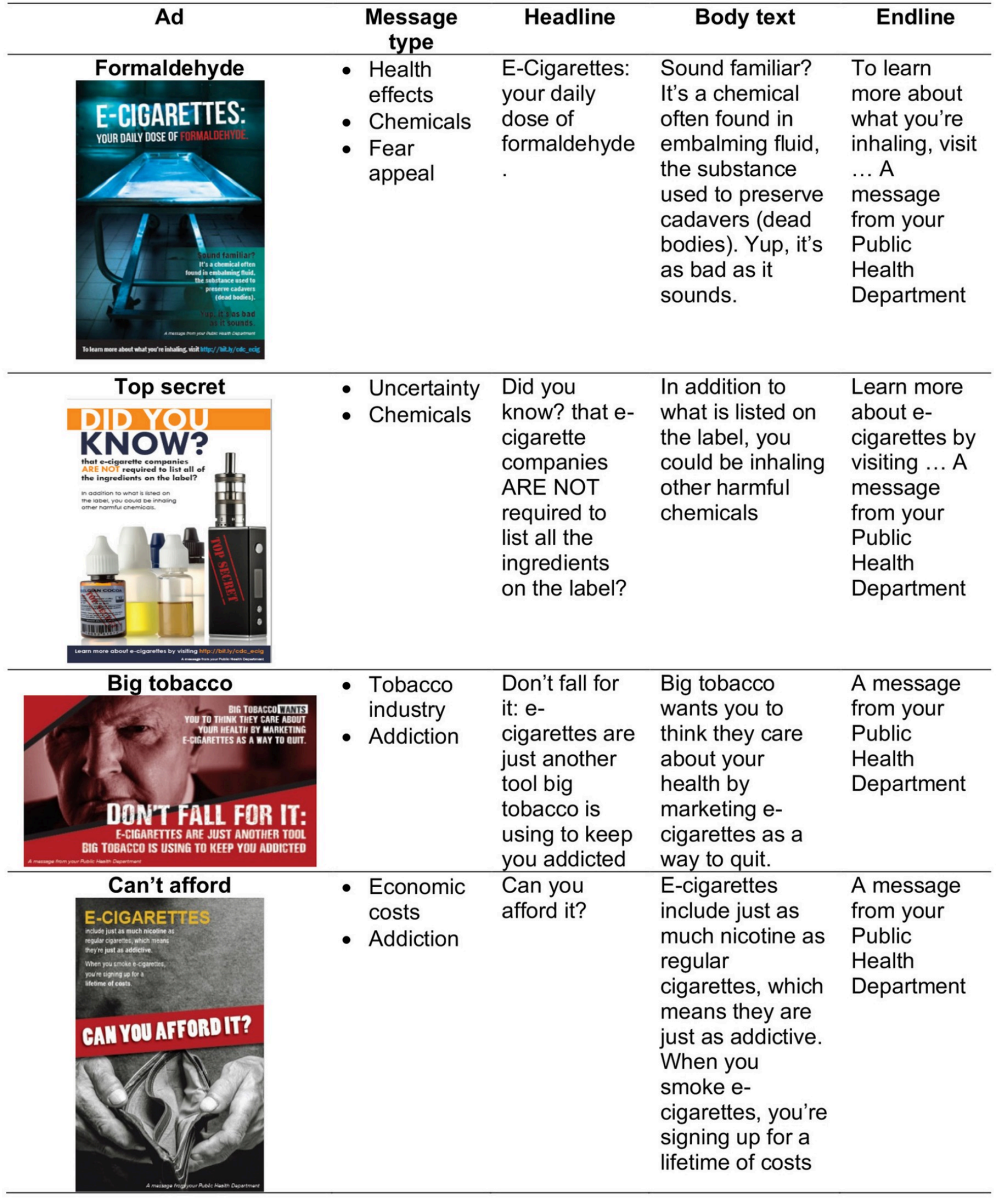
Description of e-cigarette messages used in the study.

The four messages were labelled as (1) *Formaldehyde*, (2) *Top secret*, (3) *Big tobacco*, and (4) *Can’t afford*. The *Formaldehyde* message communicated about the presence of harmful chemicals in e-cigarettes in order to increase awareness of e-liquid toxicity. *Top secret* aimed at educating the public on the uncertainty about the content of e-cigarettes and long-term health harms. *Big tobacco* provided information about the tactics employed by the tobacco companies to market their products, including making products maximally addictive, and *Can’t afford* communicated about the economic implication of e-cigarettes use, including the cost of trying to satisfy one’s craving for nicotine (see Fig 1).

### Participants

A national sample of 2,801 adult (18+ years old) non-smokers (n = 979), transitioning smokers (quit smoking in the past 2 years or currently trying to quit, n = 858), and current smokers (n = 964) was recruited from the Toluna (www.toluna-group.com) online research panel in 2018. Toluna is a survey market research company that uses multiple online recruitment strategies (e.g., web banners, website referrals, affiliate marketing, pay-per-click) to recruit eligible participants for research. Participants were purposively recruited based on their age (18+) and cigarette smoking status. All participants completed electronic informed consent. All protocols were approved by the Institutional Review Board of Georgia State University.

### Procedure

We first conducted a pilot test with 90 participants recruited by Toluna to assess the study length and refine the questionnaire. The results of the pilot study revealed that the study was feasible to conduct in under 20 minutes and the questions were understandable and easy to answer. We also reviewed qualitative feedback to the messages and did not find any issues. In the online experiment for the present study, participants began by providing information on sociodemographic factors, tobacco use, and risk perceptions of cigarettes and e-cigarettes. In addition, current smokers provided information about their intentions to quit and the number of cigarettes smoked per day. Participants were then randomized to see only one of four e-cigarette messages (see Fig 1) or a control message (bottled water ad). Similar to past studies [47–49], a bottled water advertisement was used in the control group. It was a neutral message that was not likely to have an effect on tobacco-related perceptions or intentions, but allowed to establish the effects of our messages compared to pre-existing tobacco-related perceptions and to rule out the effects of simply exposure to a message. Messages were presented online with an unlimited time for viewing. However, once participants moved on from viewing the message, they could not return to view the message again. Median time looking at the message was 14 seconds (interquartile range: 9–20 seconds). Time for each message is reported in S1 Appendix.

After viewing the message, participants were asked about their emotional responses to the message, message perceptions, self-exempting beliefs, and behavioral intentions. These outcomes were selected based on previous literature as described in the next section. At the end of the survey, all participants saw a debriefing page stating that the messages were used for research only and had not been approved by any public health or federal agency. All participants were given a quitline telephone number and referred to smoking cessation websites.

### Pre-exposure cigarette and e-cigarette use measures

#### Smoking status

Participants were asked if they had smoked 100 cigarettes in their life (*Yes* or *No*). Those who had were then asked how often they smoked now (*every day*, *some days*, or *not all*). If participants indicated “*not at all*,” then they were asked to indicate the last time they had smoked (*less than 1 month ago*, *between 1 month and 6 months ago*, *between 6 months and 1 year ago*, *between 1 year and 2 years ago*, *longer than 2 years ago*). Every day and some days smokers were asked what best represents their quitting intentions (*never expect to quit; may quit in the future*, *but not in the next 6 months; in the next 6 months; in the next month; currently trying to quit*). Participants who had smoked at least 100 cigarettes in their entire life and were currently smoking every day or some days and were not currently trying to quit were classified as current smokers. Transitioning smokers smoked at least 100 cigarettes in their entire life but had either quit smoking within the last two years or were currently trying to quit. We combined recent quitters and those trying to quit smoking because e-cigarette use in these groups may be higher than current smokers or never smokers as evidence shows high e-cigarette use prevalence in recent quitters [50], and smokers frequently use e-cigarettes to try to quit smoking [51–53]. Nonsmokers were those who had never smoked or smoked less than 100 cigarettes in their entire life.

#### E-cigarette use status

Participants were asked if they had ever used electronic cigarettes, including e-cigs, vape pens, hookah pens, personal vaporizers, moods, or JUUL (*Yes* or *No*). Ever users were then asked how often in the past 30 days they used e-cigarettes (0–30). Current e-cigarette use was defined as any past 30-day use of e-cigarettes. Former e-cigarette users had ever used e-cigarettes but not in the past 30 days. Participants who had never used e-cigarettes were classified as never e-cigarette users.

For the analyses with significant interactions between cigarette and e-cigarette use statuses, we classified participants into 9 groups: 1) never smoker & never e-cigarette user; 2) transitioning smoker & never e-cigarette user; 3) current smoker & never e-cigarette user; 4) never smoker & former e-cigarette user; 5) transitioning smoker & former e-cigarette user; 6) current smoker & former e-cigarette user; 7) never smoker & current e-cigarette user; 8) transitioning smoker & current e-cigarette user; and 9) current smoker & current e-cigarette user.

We examined whether the pattern of results differed for current e-cigarette users who were infrequent users (≤5 days), intermediate users (6–29 days), and daily users (used every day in past 30 days) [54]. Because only one interaction (for perceived effectiveness) between condition and use frequency was found, we did not report the stratified results in this paper.

### Outcomes

Guided by the Message Impact Framework which distinguishes between types of outcomes that serve as antecedents to behavioral change (e.g., message reactions, beliefs, intentions) [40], we assessed the following four blocks of outcomes (the measurement and definition of the outcomes are described in Table 1):

1. Message reactions and perceived effectiveness: informativeness [55–57], negative emotions [58, 59], reactance [60], and perceived message effectiveness [61].
2. Perception and beliefs about e-cigarettes: perceived risks of e-cigarettes [62], self-exempting beliefs [63], and support for e-cigarette regulations [64].
3. Behavioral intentions: intention to use e-cigarettes [65] and intention to communicate about health effects of e-cigarettes.
4. Intention to quit smoking among current smokers [66].

**Table 1:** Measures and definition of dependent variables.

Dependent variables and measures	Response options	Reliability
**1. Message reactions and perceived effectiveness**		
*Informativeness*	1 (not at all)– 9 (extremely)	*α* = .91
• This message was informative.		
• This message gave me a better understanding of the consequences of using e-cigarettes		
• The message was based on facts.		
• The message presented something that happens in real life.		
• The message portrayed an actual risk of e-cigarette use.		
*Negative emotions*:	1 (not at all)– 9 (extremely)	*α* = .91
While looking at the message, I felt: sad, angry, afraid, guilty, disgusted, worried, ashamed, confused		
*Reactance*:	1 (strongly disagree)– 5 (strongly agree)	*α* = .78
• This message is trying to manipulate me.		
• This message annoys me.		
• The health effects in this message are overblown.		
*Perceived effectiveness*:	1 (not at all)– 9 (extremely)	*α* = .86
• The message makes me more concerned about the health risks of e-cigarettes.		
• The message motivates me to not use e-cigarettes.		
**2. E-cigarettes beliefs and perceptions**		
*Perceived risks*:	0 (no chance)– 6 (very good chance) + I don’t know^a^	*α* = .93;
Imagine that you just began vaping e-cigarettes every day. What do you think your chances are of having each of the following happen to you if you continue to vape e-cigarettes every day?		
• Lung cancer		
• Lung disease other than lung cancer (such as COPD and emphysema)		
• Heart disease		
• Become addicted		
• Early/Premature death		
*Support for e-cigarette regulations*:	1 (strongly disagree)– 7 (strongly agree)	*α* = .89
How much do you agree or disagree that the government should:		
• ban e-cigarette promotion and advertisements in places where cigarette advertising is banned.		
• require e-cigarette packages and advertisements to carry an addiction warning.		
• require e-cigarette packages to label the amount of nicotine and other harmful ingredients.		
• regulate e-cigarettes for safety and quality standards		
*Self-exempting beliefs*:	1 (strongly disagree)– 7 (strongly agree)	*α* = .76
• E-cigarette use cannot be all that bad for you because many people who use them live long lives.		
• You can overcome the harms of e-cigarette use by doing things like eating healthy food and exercising regularly.		
• You have got to die of something, so why not enjoy yourself and use e-cigarette.		
• Everything causes cancer these days.		
**3. Behavioral intentions**		
*Intentions to use e-cigarettes in the future*:	1 (not at all)– 9 (extremely)	
How open are you to trying e-cigarettes in the future?		
*Willingness to communicate about health effects of e-cigarettes*	1 (not at all)– 9 (extremely)	*α* = .87
• Share messages about health risks of e-cigarettes on social media.		
• Talk to your friends about health risks of e-cigarettes.		
*Intention to quit smoking*	0 (very definitely no)– 10 (very definitely yes)	*N/A*
How much do you intend to quit smoking in the next 6 months?		

^a^ “I don’t know” responses were treated as missing.

### Covariates

We included sex, age, race, education, cigarette smoking, and e-cigarette use as covariates in the analyses. Sex was categorized as male and female; age was categorized into 18–29 years, 30–44 years, 45–59 years, and ≥60 years; race was categorized into white, black, and other; and education was classified as below high school, high school, some college, and bachelor’s or higher.

### Statistical analysis

We performed descriptive analyses to detail the characteristics of study participants. Multivariate analyses of variance (MANOVA) were conducted to examine the effect of the experimental condition on the three blocks of outcomes: message reactions and perceived effectiveness, e-cigarettes beliefs and perceptions, and behavioral intentions. Following the significant multivariate effect of condition, univariate analyses estimated adjusted means for each experimental condition for each outcome. For the message reactions and perceived effectiveness block of outcomes, we excluded the control group as a comparator because reactions to the control message (bottled water ad) were not relevant. Univariate analysis was conducted to assess the impact of the messages on current smokers’ intention to quit smoking in the next 6 months. In all analyses, the *p*-values were adjusted for the multiple pairwise comparisons using the Bonferroni correction method. Two-way and three-way interactions between the experimental condition, smoking status, and e-cigarette use status were examined for all outcomes except the intention to quit outcome. The smoking and e-cigarette use interaction assessment allowed us to examine the impacts of different combinations of smoking and e-cigarette use status on the study outcomes. For the outcome variables in which significant interaction was observed, the analyses were stratified accordingly. In addition, a supplementary analysis tested interaction between transitioning smoker status (recent quitters vs trying to quit) and condition but did not find any significant interaction in any of the outcomes (*Ps*>0.1) (Data not shown). All estimates were adjusted for sex, age, education, and race. In addition to the demographic factors, smoking status was adjusted for in the analysis stratified by e-cigarette use status. Analyses using the total sample also adjusted for smoking status and e-cigarette use status. Cohen’s d was calculated as differences in means divided by the corresponding pooled standard deviation. All analyses were completed in SAS version 9.4 (SAS Institute, Cary, NC, USA) in 2019.

## Results

Of the total sample (n = 2801), 35.0% were non-smokers (n = 979), 30.6% were transitioning smokers (quit smoking in the past 2 years or currently trying to quit, n = 858), 34.4% were current smokers (n = 964), 15.6% were former e-cigarette users (n = 436), and 23.9% were current e-cigarette users (n = 669). The sample was 18–95 years old (mean = 48 years, SD = 17 years), 50.7% female, 78.9% white, and 36.6% had bachelor’s degree or higher (Table 2).

**Table 2:** Characteristics of study participants overall and by the message condition.

Variable	Total (n = 2801)	Formaldehyde (n = 564)	Top secret (n = 555)	Big tobacco (n = 586)	Can’t afford (n = 544)	Control (n = 552)
**Sex**						
Male	49.3	49.1	47.0	48.6	50.4	51.5
Female	50.7	50.9	53.0	51.4	49.6	48.6
**Age**						
18–29 years	19.4	21.6	19.5	18.8	19.7	17.2
30–44 years	24.2	22.0	26.3	25.9	21.3	25.4
45–59 years	25.4	26.6	24.3	27.3	24.6	23.7
60 years or older	31.1	29.8	29.9	28.0	34.4	33.7
**Education**						
<High school	6.5	5.5	7.9	6.7	6.6	5.6
High school	24.5	23.2	24.9	25.6	24.6	24.3
Some college	32.5	33.2	33.2	32.3	32.2	31.5
Bachelor’s or higher	36.6	38.1	34.1	35.5	36.6	38.6
**Race**						
White	78.9	80.1	78.0	78.3	80.9	77.4
Black	11.3	9.9	11.9	12.0	11.0	11.6
Other	9.8	9.9	10.1	9.7	8.1	11.1
**Smoking status**						
Non-smoker	35.0	36.9	34.4	33.8	35.3	34.4
Transitioning smoker	30.6	28.9	31.9	34.5	28.9	28.8
Current smoker	34.4	34.2	33.7	31.7	35.9	36.8
**E-cigarette use status**						
Never user	60.6	60.6	60.5	58.9	62.0	60.9
Former user	15.6	16.1	14.4	17.1	15.1	15.0
Current user	23.9	23.2	25.1	24.1	23.0	24.1

### Message reactions and perceived effectiveness

There was a significant multivariate effect of condition on the four outcomes within this block (Wilks’ λ = 0.79, *P* < .01). Significant interaction between condition and e-cigarette use status was observed for only perceived effectiveness (*P*< .01). All other interactions were not significant.

#### Informativeness

Participants perceived *Formaldehyde* as more informative than all other e-cigarette messages. *Big tobacco* was rated as least informative, although its score did not differ significantly from that of *Top secret* (Tables 3 & 4).

**Table 3:** Impact of condition on dependent variables in the total sample and by smoking and e-cigarette use status.

Outcome	Formaldehyde LSM (95% CI)	Top secret LSM (95% CI)	Big tobacco LSM (95% CI)	Can’t afford LSM (95% CI)	Control LSM (95% CI)
**Message reactions and effectiveness**					
*Informativeness*					
Total sample (n = 2249)	6.5 (6.2, 6.7)^a^	5.8 (5.6, 6.1)^bc^	5.5 (5.3, 5.8)^c^	6.1 (5.9, 6.3)^b^	n/a
*Reactance*					
Total sample (n = 2249)	2.5 (2.4, 2.6)^b^	2.5 (2.4, 2.7)^b^	2.7 (2.6, 2.9)^a^	2.5 (2.4, 2.6)^b^	n/a
*Negative emotions*					
Total sample (n = 2249)	4.1 (3.9, 4.3)^a^	3.5 (3.3, 3.7)^b^	3.4 (3.2, 3.6)^b^	3.2 (3.0, 3.4)^b^	n/a
*Perceived Effectiveness*					
Total sample (n = 2249)	6.7 (6.4, 6.9)^a^	6.3 (6.0, 6.5)^b^	5.7 (5.4, 5.9)^c^	6.1 (5.8, 6.3)^bc^	n/a
Never e-cigarette user (n = 1360)	6.9 (6.6, 7.2)^a^	6.7 (6.4, 7.0)^a^	6.2 (5.9, 6.5)^b^	6.7 (6.4, 7.0)^a^	n/a
Former e-cigarette user (n = 353)	6.9 (6.2, 7.5)^a^	6.3 (5.6, 7.0)^ab^	5.4 (4.7, 6.0)^bc^	4.8 (4.1, 5.5)^c^	n/a
Current e-cigarette user (n = 536)	6.5 (5.9, 7.0)^a^	5.7 (5.1, 6.3)^ab^	5.5 (4.9, 6.1)^b^	5.9 (5.3, 6.5)^ab^	n/a
**E-cigarettes beliefs and perceptions**					
*Perceived risk of e-cigarettes*					
Total sample (n = 2344)	4.4 (4.2, 4.6)^a^	4.1 (3.9, 4.2)^b^	4.2 (4.0, 4.3)^abc^	4.3 (4.1, 4.5)^abc^	3.8 (3.7, 4.0)^bd^
*Support for e-cigarette control*					
Total sample (n = 2801)	5.6 (5.4, 5.8)^a^	5.5 (5.4, 5.7)^a^	5.5 (5.3, 5.6)^a^	5.4 (5.3, 5.6)^ab^	5.2 (5.1, 5.4)^b^
*Self-exempting beliefs*					
Total sample (n = 2801)	3.5 (3.4, 3.7)^b^	3.6 (3.5, 3.7)^ab^	3.6 (3.5, 3.7)^b^	3.6 (3.5, 3.8)^ab^	3.8 (3.7, 4.0)^a^
**Behavioral intentions**					
*Intention to use e-cigarettes*					
Never smoker & never e-cigarette user (n = 878)	1.6 (1.4, 1.9)^a^	1.6 (1.3, 1.8)^a^	1.9 (1.6, 2.1)^a^	1.6 (1.4, 1.9)^a^	1.7 (1.4, 1.9)^a^
Transitioning smoker & never e-cigarette user (n = 342)	2.0 (1.3, 2.7)^b^	3.1 (2.5, 3.8)^ab^	3.1 (2.5, 3.7)^ab^	2.9 (2.2, 3.6)^ab^	3.5 (2.8, 4.1)^a^
Current smoker & never e-cigarette user (n = 476)	2.8 (2.3, 3.4)^a^	3.2 (2.6, 3.8)^a^	3.3 (2.7, 3.9)^a^	3.1 (2.5, 3.7)^a^	3.5 (2.9, 4.0)^a^
Never smoker & former e-cigarette user (n = 51)	1.2 (-0.6, 2.9)^a^	2.1 (0.2, 4.1)^a^	2.2 (0.0, 4.4)^a^	3.3 (1.7, 5.0)^a^	1.0 (-0.9, 2.9)^a^
Transitioning smoker & former e-cigarette user (n = 168)	2.3 (1.4, 3.3)^a^	2.8 (1.8, 3.8)^a^	2.8 (2.0, 3.7)^a^	3.3 (2.3, 4.3)^a^	3.1 (2.0, 4.2)^a^
Current smoker & former e-cigarette user (n = 217)	3.3 (2.3, 4.3)^b^	3.7 (2.7, 4.7)^b^	4.6 (3.8, 5.5)^ab^	4.5 (3.5, 5.6)^ab^	5.3 (4.4, 6.2)^a^
Never smoker & current e-cigarette user (n = 50)	6.0 (3.7, 8.2)^a^	6.3 (3.8, 8.7)^a^	6.1 (3.6, 8.7)^a^	4.6 (1.7, 7.5)^a^	5.9 (3.6, 8.2)^a^
Transitioning smoker & current e-cigarette user (n = 348)	5.2 (4.4, 6.0)^b^	6.1 (5.3, 6.9)^ab^	5.8 (5.1, 6.6)^ab^	5.8 (4.9, 6.6)^ab^	6.7 (6.0, 7.5)^a^
Current smoker & current e-cigarette user (n = 271)	4.1 (3.3, 4.9)^b^	5.1 (4.3, 5.9)^ab^	5.9 (5.1, 6.8)^a^	5.7 (5.0, 6.5)^a^	6.0 (5.2, 6.8)^a^
*Willingness to communicate about the risk of e-cigarette*					
Never smoker/never e-cigarette user (n = 878)	3.1 (2.8, 3.4)^a^	3.2 (2.9, 3.5)^a^	2.9 (2.6, 3.2)^a^	3.3 (3.0, 3.6)^a^	2.9 (2.6, 3.3)^a^
Transitioning smoker & never e-cigarette user (n = 342)	4.1 (3.6, 4.6)^a^	3.1 (2.7, 3.6)^b^	3.8 (3.4, 4.3)^ab^	3.6 (3.1, 4.1)^ab^	3.8 (3.3, 4.3)^ab^
Current smoker & never e-cigarette user (n = 476)	3.1 (2.7, 3.5)^ab^	3.2 (2.8, 3.6)^a^	2.6 (2.2, 3.1)^ab^	2.6 (2.2, 3.0)^ab^	2.5 (2.1, 2.8)^b^
Never smoker & former e-cigarette user (n = 51)	4.5 (3.4, 5.5)^a^	3.0 (1.8, 4.2)^ab^	3.3 (1.9, 4.7)^ab^	2.4 (1.4, 3.4)^b^	4.1 (2.9, 5.3)^ab^
Transitioning smoker & former e-cigarette user (n = 168)	3.4 (2.6, 4.1)^a^	2.7 (1.9, 3.4)^a^	3.2 (2.5, 3.9)^a^	2.8 (2.0, 3.6)^a^	2.5 (1.6, 3.4)^a^
Current smoker & former e-cigarette user (n = 217)	2.5 (1.9, 3.1)^a^	2.5 (1.8, 3.2)^a^	2.2 (1.6, 2.8)^a^	2.4 (1.7, 3.1)^a^	2.1 (1.5, 2.7)^a^
Never smoker & current e-cigarette user (n = 50)	3.3 (1.9, 4.8)^a^	3.0 (1.4, 4.6)^a^	3.7 (2.0, 5.3)^a^	4.1 (2.2, 6.0)^a^	2.4 (0.9, 3.9)^a^
Transitioning smoker & current e-cigarette user (n = 348)	3.4 (2.8, 3.9)^a^	2.9 (2.4, 3.5)^a^	3.4 (2.8, 3.9)^a^	3.6 (3.0, 4.2)^a^	3.4 (2.9, 3.9)^a^
Current smoker & current e-cigarette user (n = 271)	3.2 (2.6, 3.7)^a^	3.1 (2.6, 3.7)^a^	2.9 (2.3, 3.4)^a^	3.2 (2.6, 3.7)^a^	2.7 (2.1, 3.3)^a^
**Intention to quit smoking**					
All current smokers who are not trying to quit	5.7 (5.2, 6.3)^a^	5.8 (5.2, 6.3)^a^	5.9 (5.3, 6.4)^a^	5.7 (5.2, 6.3)^a^	5.7 (5.2, 6.2)^a^

LSM, least square means; CI, Confidence intervals. Estimates are from MANOVAs in SAS.

All estimates were adjusted for sex, age, race, and educational level. Smoking and e-cigarette status were also adjusted for in the overall estimates, and smoking use status was also adjusted for the stratified analyses. Estimates with different superscripts in each row are significantly different (p<0.05).

**Table 4:** Impact of condition on dependent variables in the total sample and by smoking and e-cigarette use status—Cohen’s d effect size.

Outcome	Cohen’ s d			
	Formaldehyde vs Control	Top Secret vs Control	Big Tobacco vs Control	Can’t Afford vs Control
**E-cigarettes beliefs and perceptions**				
*Perceived risk of e-cigarettes*				
Total sample (n = 2344)	0.38	0.16	0.22	0.31
*Support for e-cigarette control*				
Total sample (n = 2801)	0.27	0.22	0.20	0.14
*Self-exempting beliefs*				
Total sample (n = 2801)	-0.19	-0.15	-0.16	-0.12
**Behavioral intentions**				
*Intention to use e-cigarettes*				
Never smoker/never e-cigarette user (n = 878)	-0.04	-0.07	0.15	-0.05
Transitioning smoker & never e-cigarette user (n = 342)	-0.60	-0.14	-0.15	-0.23
Current smoker & never e-cigarette user (n = 476)	-0.31	-0.12	-0.08	-0.17
Never smoker & former e-cigarette user (n = 51)	0.06	0.49	0.54	1.05
Transitioning smoker & former e-cigarette user (n = 168)	-0.35	-0.15	-0.14	0.06
Current smoker & former e-cigarette user (n = 217)	-0.86	-0.69	-0.27	-0.32
Never smoker & current e-cigarette user (n = 50)	0.02	0.14	0.08	-0.46
Transitioning smoker & current e-cigarette user (n = 348)	-0.59	-0.24	-0.33	-0.35
Current smoker & current e-cigarette user (n = 271)	-0.81	-0.38	-0.03	-0.11
*Willingness to communicate about the risk of e-cigarette*				
Never smoker/never e-cigarette user (n = 878)	0.11	0.15	-0.04	0.21
Transitioning smoker & never e-cigarette user (n = 342)	0.19	-0.40	0.04	-0.11
Current smoker & never e-cigarette user (n = 476)	0.39	0.46	0.11	0.09
Never smoker & former e-cigarette user (n = 51)	0.25	-0.80	-0.59	-1.22
Transitioning smoker & former e-cigarette user (n = 168)	0.47	0.10	0.40	0.15
Current smoker & former e-cigarette user (n = 217)	0.26	0.27	0.06	0.19
Never smoker & current e-cigarette user (n = 50)	0.52	0.31	0.71	0.95
Transitioning smoker & current e-cigarette user (n = 348)	0.004	-0.25	-0.01	0.14
Current smoker & current e-cigarette user (n = 271)	0.29	0.25	0.09	0.29
*Intention to quit smoking*				
All current smokers who are not trying to quit	0.02	0.04	0.05	0.03

Note: Negative value indicates that the control mean was greater than the mean in the experimental condition. Adjusted means were obtained from MANOVAs in SAS. The means were adjusted for sex, age, race, and educational level. Smoking and e-cigarette status were also adjusted for in the overall estimates, and smoking use status was also adjusted for the stratified analyses. Cohen’s d was calculated by dividing mean differences by the corresponding pooled standard deviation. Cohen’s rule of thumb standard for effect sizes is: Small: d = 0.2, medium: d = 0.5, large: d = 0.8.

#### Reactance

Reactance to the messages was highest in *Big tobacco* but did not differ between the other three messages (Tables 3 & 4).

#### Negative emotions

*Formaldehyde* evoked significantly higher levels of negative emotions compared to the other three messages. The other messages were rated similar in negative emotions (Tables 3 & 4).

#### Perceived effectiveness

Because the interaction between condition and e-cigarette use was significant for perceived effectiveness, we examined e-cigarette use groups separately. All e-cigarette use groups consistently rated *Formaldehyde* as the most effective. However, while never and current e-cigarette users rated *Big tobacco* as the least effective among the e-cigarette messages, former e-cigarette users rated *Can’t afford* as least effective (Tables 3 & 4).

### E-cigarettes beliefs and perceptions

Condition showed significant effects on perceived risk of e-cigarettes, support for e-cigarette regulations and self-exempting beliefs regarding e-cigarettes in the MANOVA (Wilks’ λ = 0.97, *P* < .01). Condition by smoking status interaction was not significant for all three outcomes. In the total sample, all e-cigarette messages, except *Top secret*, elicited higher e-cigarette risk perception than control. Higher support for e-cigarette regulations was observed in all e-cigarette messages, except *Can’t afford*, compared to the control group. Additionally, participants who saw *Formaldehyde* and *Big Tobacco* had lower self-exempting beliefs compared to the control group. Self-exempting belief scores did not differ significantly among the e-cigarette messages (Tables 3 & 4).

### Behavioral intentions

Condition showed significant effects on the two outcome variables in the MANOVA (Wilks’ λ = 0.97, *P* < .01). There was a significant condition*smoking status*e-cigarette use status interaction in both intentions to use e-cigarettes and willingness to communicate about the health risk of e-cigarette use (*Ps* = .04), so the analyses were stratified accordingly in both outcomes.

#### Intention to use e-cigarettes

**Fig 2:**
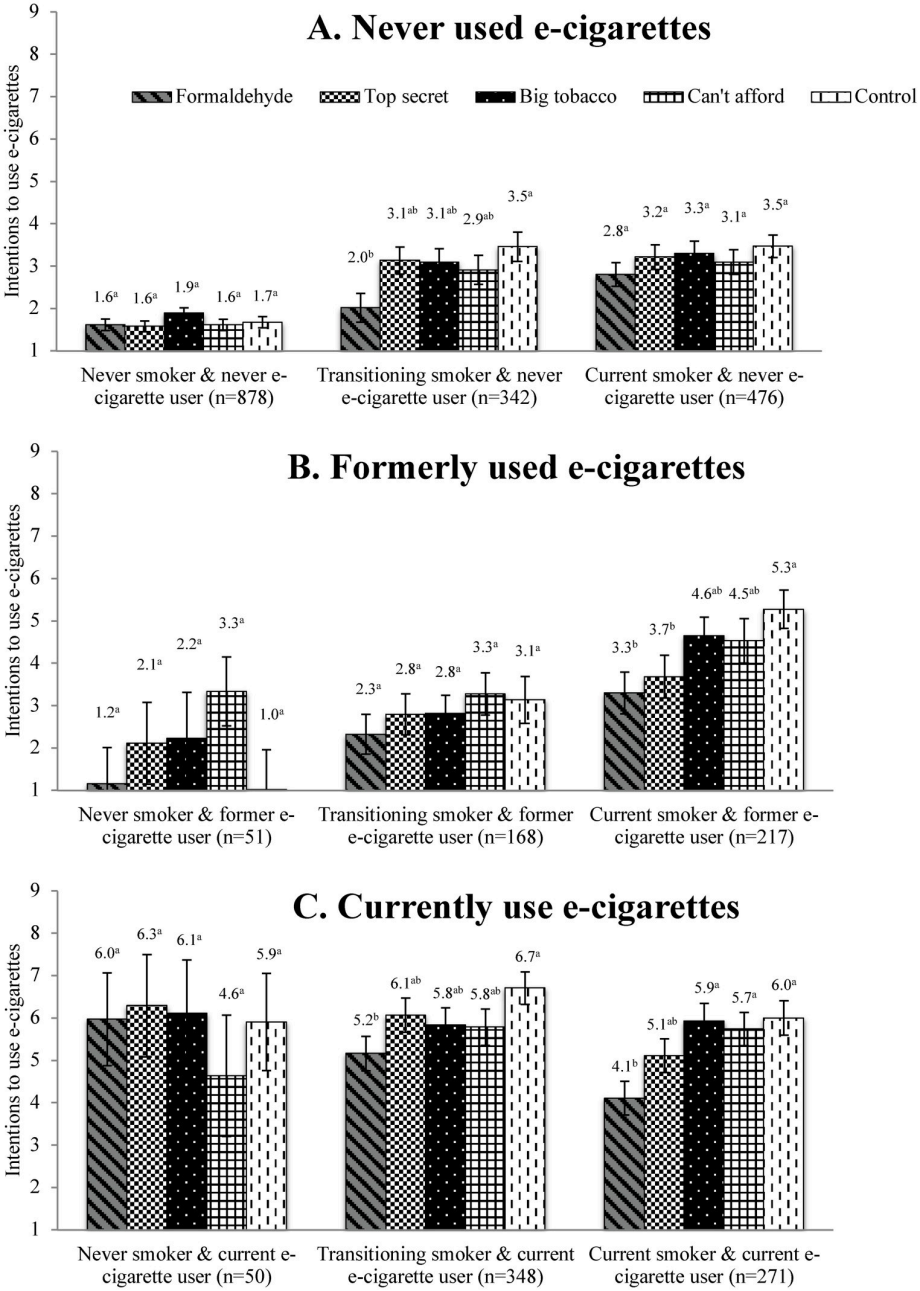
Intentions to use e-cigarettes among never, transitioning, and current smokers who never, formerly, and currently use e-cigarettes. Estimates with different superscripts are significantly different (p<0.05).

Participants in the *Formaldehyde* condition had lower intentions to use e-cigarettes compared to the control among transitioning smokers & never e-cigarette user, current smokers & former e-cigarette users, transitioning smokers & current e-cigarette users, and current smokers/current e-cigarette users. Condition showed no significant effect on intentions to use e-cigarettes in never smokers & never e-cigarette users, current smokers & never e-cigarette users, never smokers & former e-cigarette users, transitioning smokers & former e-cigarette users, and never smokers & current e-cigarette users (Tables 3 & 4 and Fig 2).

#### Willingness to communicate about health risks of e-cigarettes

In general, the e-cigarette messages did not differ significantly from the control and all messages were rated similarly in all groups except among transitioning smokers & never e-cigarette users, current smokers & never e-cigarette users, and never smokers & former e-cigarette users. While transitioning smokers & never e-cigarette users who saw *Top secret* were least willing to communicate about e-cigarettes, current smokers & never e-cigarette users who saw the control message had the lowest intentions to communicate about e-cigarettes (Tables 3 & 4).

### Intention to quit smoking

Condition did not have a significant effect on current smokers’ intention to quit smoking (Tables 3 & 4).

## Discussion

The upsurge of e-cigarette use [67–69], particularly among never smokers [70, 71], makes it imperative to develop effective messages aimed at preventing e-cigarette use. At least 14 countries have implemented warning labels on e-cigarettes [72], and as more evidence on the harms of e-cigarettes continues to emerge, the need for effective messages about harms of e-cigarettes will likely increase. We developed and tested four e-cigarette messages that used themes similar to the ones used in antismoking communications. The results showed that the message that indicated that e-cigarettes contain formaldehyde was perceived as the most informative and effective. It also elicited the highest levels of negative emotions. However, several messages performed equally well on measures of actual effectiveness (perceptions of risk, support for regulations, and behavioral intentions). Specifically, we observed increased perceived risk of e-cigarettes in all messages but *Top Secret*, and increased support for e-cigarette control in all messages but *Can’t Afford*. A significant decrease in intentions to use e-cigarettes was observed among transitioning smokers who had never used e-cigarettes, current smokers who were former e-cigarette users, transitioning smokers who currently used e-cigarettes, and current smokers who were current e-cigarette users who saw *Formaldehyde*. *Top secret* was associated with decreased intention to use e-cigarettes in current smokers who were former e-cigarette users. The effect sizes we observed for intentions to use were generally small to medium (Cohen’s d = 0.2–0.5); these sizes are comparable to average effect sizes in communication research [73]. *Formaldehyde* elicited the largest effect sizes for behavioral intentions (except in user groups with small sample size), which is consistent with it being the message that evoked the highest levels of negative emotions and was perceived as the most effective across all categories of users. These findings indicate that while messages about *Formaldehyde* in e-cigarettes might be viewed by consumers as the most effective in deterring the use of e-cigarettes, these and also other messages might have an impact on actual beliefs, perceptions, and intentions. Thus, a variety of messages can be used to derive maximum benefits from health communications around e-cigarettes.

Currently, the FDA requires manufacturers to warn consumers about one ingredient in e-cigarettes (nicotine), although the agency acknowledges that the effect of a single label will wear off over time [31]. FDA is poised to conduct research on warning labels to ensure that there are efficacious warning labels that offer public education on the contents of e-cigarettes [31]. Our findings suggest the importance of communicating about the contents of e-cigarettes. A qualitative study has reported that participants preferred information on actual ingredients and toxic chemicals in e-cigarettes to information about general health effects of e-cigarettes [21]. In our qualitative study of comparative risk messages, we also observed that the mentioning of specific ingredients in e-cigarettes, including nicotine and formaldehyde, made participants perceive e-cigarettes as very harmful, and they were skeptical that e-cigarettes are less harmful than cigarettes. Additionally, comparative risk messages that compared toxic chemicals in cigarettes and e-cigarettes were consistently viewed as factual [18]. These findings, together with our results showing significant impact of *Formaldehyde* on the outcomes, suggest the need to communicate about contents of e-cigarettes, including the presence of toxic chemicals such as formaldehyde. Therefore, it is important to include information about toxic chemical contents of e-cigarettes in e-cigarette warning labels and public education messages.

Besides potential health harms of e-cigarettes, e-cigarette use also carries an economic burden for users. Consumers who become addicted to nicotine through e-cigarette use will spend money to satisfy the addiction. This needs to be communicated to the public, especially nonsmokers who are not yet addicted to nicotine. In this study, one of the messages, *Can’t afford*, focused on economic effects of e-cigarette use. We observed that this message significantly impacted perceived risk of e-cigarettes but did not differ from the control in other outcomes. This message had the largest effect size for intentions to use e-cigarettes (Cohen’s *d* = -0.46) for the group who would potentially benefit from e-cigarette prevention messages the most—never smokers & current e-cigarette users. Although this effect was not significant due to the small group size (n = 50), more studies are needed to assess the impacts of messages communicating economic costs of e-cigarette use on intentions and actual e-cigarette use behavior.

Importantly, compared to controls, all messages did not affect smokers’ intention to quit smoking (intentions did not decrease or increase). Some e-cigarette advocates argue that messages communicating about harms of e-cigarettes are unethical because they will decrease smokers’ intentions to quit smoking (by using e-cigarettes) and will promote continued smoking. We found that among current smokers who are not currently trying to quit, the intentions to quit smoking in the next 6 months were between 5.7 and 5.9 across all the conditions (on a 9-point scale). This provides preliminary evidence that messages about harms of e-cigarettes may not negatively impact quitting intentions among smokers.

The group that can particularly benefit from messages discouraging e-cigarette use are never smokers & current e-cigarette users. However, we found no statistically significant difference in the intentions to use e-cigarettes for different messages among this group. This lack of significance can be explained by the small group size (n = 50). Nonetheless, it is noteworthy that never smokers/current e-cigarette users who saw the message focused on the financial costs of e-cigarette use, had lower intentions compared to the control (Cohen’s d = -0.46). Future studies should recruit larger sample of never smokers/current e-cigarette users and investigate themes focused on financial loss and possibly other topics, such as social norms or harm to others [74].

Some might argue that reducing the intentions to use e-cigarettes among current smokers is an undesirable outcome. We agree that if smokers completely switched to e-cigarettes, they would potentially reduce the health harms from smoking. However, patterns of e-cigarette use indicate that complete switching is rare, dual use is common [75], and among dual users, daily smoking and occasional e-cigarette use is the most prevalent pattern [76]. It has also been demonstrated that over time the population of smokers in the US and Europe is softening with more smokers making quit attempts and smoking fewer cigarettes [77, 78]. In the US, what differentiates smokers who never plan to quit with those with quitting intentions is not the perceived harms of e-cigarettes, but lower perceived harms of combusted cigarettes [79]. Therefore, instead of trying to make smokers switch to e-cigarettes, efforts should continue to motivate their complete cessation.

There are limitations that need to be considered in this study. We are unable to state how the findings will translate into actual behavior changes. Future studies should examine whether e-cigarette health messages can result in changing behavior. Our messages differed not only on theme, but also on the execution, color, text size and font, and use of specific images, which could also explain the differences we found. Future studies should examine individual message features to dissociate their effects. The study examined exposure effects from a single message at one point in time. Future studies should test exposure to multiple messages over time and vary the number of messages and duration of exposure. These alternative designs might help disentangle acute exposure effects from habituation effects that might occur due to repeated exposure. Although we selected the participants to be similar in demographic characteristics to the adult population in the US, the sample was not representative of adults in the US. This limits the generalizability of the findings. Finally, the item measuring intentions for e-cigarette use “*How open are you to trying e-cigarettes in the future*?” was more appropriate for never users, but we used it for current users as well in order to be able to compare across the groups. Future studies should measure current users’ behavioral intentions with questions about using rather than trying e-cigarettes in the future.

## Conclusions

The results of this study suggest that e-cigarette messages that use antismoking message strategies may be effective in communicating potential risks of e-cigarettes. The results indicate that because different messages influenced different outcomes, there is a need to use multiple e-cigarette messages to communicate with the public. This study informs future education campaigns intended to educate the public about the harms of e-cigarettes.

## Supporting information

S1 Appendix Median time and Interquartile Range (IQR) spent looking at each message. (DOCX)
