# Supplementary material for: An experimental study of messages communicating potential harms of electronic cigarettes
Source: PLoS One. 2020 Oct 21;15(10):e0240611. doi: 10.1371/journal.pone.0240611 (PMC7577451; doi:10.1371/journal.pone.0240611)
Supplement: S1 Appendix — (DOCX) [file pone.0240611.s001.docx]

**S1 Appendix.** Median Time and Interquartile Range (IQR) Spent Looking at Each Message

| **Message** | **Median** | **IQR** |
| --- | --- | --- |
| Formaldehyde | 15 | 10 - 23 |
| Top Secret | 13 | 9 - 19 |
| Big Tobacco | 14 | 10 - 22 |
| Can’t Afford | 13 | 9 - 19 |
| Control | 13 | 8 - 20 |
